# Community health workers at the dawn of a new era: 2. Planning, coordination, and partnerships

**DOI:** 10.1186/s12961-021-00753-7

**Published:** 2021-10-12

**Authors:** Muhammad Mahmood Afzal, George W. Pariyo, Zohra S. Lassi, Henry B. Perry

**Affiliations:** 1 Global Health and Medical Complex, Polyversity International, Lahore, Pakistan; 2 Health Systems Program, Department of International Health, Johns Hopkins Bloomberg School of Public Health, Baltimore, MD USA; 3 Robinson Research Institute, Faculty of Health and Medical Sciences, The University of Adelaide, Adelaide, Australia

**Keywords:** Health workforce, Health system, Health systems planning, Community health workers, Coordination, Partnerships, Essential health services, Sustainable Development Goals, Universal Health Coverage, Maternal and child health

## Abstract

**Background:**

Community health workers (CHWs) play a critical role in grassroots healthcare and are essential for achieving the health-related Sustainable Development Goals. While there is a critical shortage of essential health workers in low- and middle-income countries, WHO and international partners have reached a consensus on the need to expand and strengthen CHW programmes as a key element in achieving Universal Health Coverage (UHC). The COVID-19 pandemic has further revealed that emerging health challenges require quick local responses such as those utilizing CHWs. This is the second paper of our 11-paper supplement, “Community health workers at the dawn of a new era”. Our objective here is to highlight questions, challenges, and strategies for stakeholders to consider while planning the introduction, expansion, or strengthening of a large-scale CHW programme and the complex array of coordination and partnerships that need to be considered.

**Methods:**

The authors draw on the outcomes of discussions during key consultations with various government leaders and experts from across policy, implementation, research, and development organizations in which the authors have engaged in the past decade. These include global consultations on CHWs and global forums on human resources for health (HRH) conferences between 2010 and 2014 (Montreux, Bangkok, Recife, Washington DC). They also build on the authors’ direct involvement with the Global Health Workforce Alliance.

**Results:**

Weak health systems, poor planning, lack of coordination, and failed partnerships have produced lacklustre CHW programmes in countries. This paper highlights the three issues that are generally agreed as being critical to the long-term effectiveness of national CHW programmes—planning, coordination, and partnerships. Mechanisms are available in many countries such as the UHC2030 (formerly International Health Partnership), country coordinating mechanisms (CCMs), and those focusing on the health workforce such as the national Human Resources for Health Observatory and the Country Coordination and Facilitation (CCF) initiatives introduced by the Global Health Workforce Alliance.

**Conclusion:**

It is imperative to integrate CHW initiatives into formal health systems. Multidimensional interventions and multisectoral partnerships are required to holistically address the challenges at national and local levels, thereby ensuring synergy among the actions of partners and stakeholders. In order to establish robust and institutionalized processes, coordination is required to provide a workable platform and conducive environment, engaging all partners and stakeholders to yield tangible results.

Key message box 1: summary **Key findings**

Community health worker (CHW) programmes in many countries have generally been shown to be effective in improving health. However, they are often hindered by:

- weak health systems,
- poor planning,
- lack of coordination, and
- failed partnerships that have produced lacklustre CHW programmes.

The development, strengthening, and governance of CHW programmes need to take into account the country’s health system’s socioeconomic and political context and tailor solutions to the country’s political economy.

**Key implications**

The key principles on which there is broad agreement in making CHW programmes effective include strengthening:

- planning,
- coordination, and
- partnerships.

## Background

Following the era of the Millennium Development Goals (MDGs), which ended in 2015, the world adopted the Sustainable Development Goals (SDGs) [1]. At this historical juncture, WHO began a renewed focus on primary healthcare (PHC) and promoted Universal Health Coverage (UHC) as a tool to achieve the health-related SDGs. The critical shortages of the essential health workforce in low- and middle-income countries (LMICs) pose a serious obstacle to achieving the health-related SDGs [2]. In addition, other related challenges such as the geographic maldistribution of human resources for health (HRH), poor working conditions, low rates of retention, and weak management systems exacerbate health system weaknesses in providing essential health services at the local level [3, 4].

As has been repeatedly emphasized, CHWs “are not a panacea for weak health systems” [5]. Nonetheless, the growing awareness of the potential of CHWs to address many critical gaps in health systems throughout the world and the critical role they can play in responding to health emergencies such as the COVID-19 pandemic has now placed national CHW programmes at the dawn of a new era.

Thus, it is an appropriate moment to step back and examine three broad issues that are critical to the long-term effectiveness of national CHW programmes—planning, coordination, and partnerships. Governance and financing are equally important topics that are addressed in separate papers in this current series [6, 7]. In 2014, together with other colleagues, we addressed these issues in the chapters of a larger volume entitled *Developing and Strengthening Community Health Worker Programs at Scale: A Reference Guide and Case Studies for Program Managers and Policymakers* [8–10]. It was our intent at that time to follow up promptly with journal articles along the same themes [11], but other priorities intervened. More recently, one of us (HP) led the publication of the most comprehensive and current descriptions available for 29 national CHW programmes in a book entitled *Health for the People: National Community Health Worker Programs from Afghanistan to Zimbabwe* [12]*.*

This paper, the second in the current series of 11 articles on national CHW programmes entitled “Community Health Workers at the Dawn of a New Era”, builds on those earlier efforts. It seeks to draw out the principles that continue to have relevance for the new era that CHW programmes are entering. Learning from past experience has critical relevance in the domain of planning, coordination, and partnerships. A previous article coauthored by one of us (HP) describes the experience of the national Village Health Guides programme in India (1977–2002), whose ultimate demise was attributable to poor planning, lack of coordination, and failed partnerships [13].

The introduction of any new health worker category into a health system is a complicated task because health systems themselves are complex and have many moving, interlocking subsystems. Introducing a new CHW cadre, as opposed to a higher-level category of health worker, is even more complicated because it also directly involves communities and their relationship with this new frontline health worker (FLHW). For this reason, planning, coordination, and partnerships are vital at the time of the introduction of a national-level CHW programme as well as when major changes are being envisioned for the programme. How to do this effectively when so many LMICs have weak national governance and management systems is a particular challenge.

The emergent health challenges such as the outbreaks of severe acute respiratory syndrome (SARS), Middle East respiratory syndrome (MERS), and more recently severe acute respiratory syndrome coronavirus 2 (SARS-CoV-2), the virus which has caused the COVID-19 pandemic, have exposed serious weaknesses of the health systems throughout the world. Among these are shortages of well-trained and skilful essential health workers at the front line to assist with urgent tasks such as promotion of appropriate protective behaviours, contact tracing, and immunization of the entire population. It is globally acknowledged that no single actor or organization can improve the health workforce situation in any given country. Therefore, multidimensional interventions and multisectoral partnerships are essential, at both national and local levels. The challenge is how to develop multisectoral and multi-stakeholder partnerships for planning, coordination, and governance, and attain synergy among the actions of partners and stakeholders.

In 2010, Bhutta, Lassi, Pariyo, and Huicho published a comprehensive review of eight national CHW programmes: *Global Experience of Community Health Workers for Delivery of Health Related Millennium Developmental Goals: A Systematic Review, Country Case Studies, and Recommendations for Integration into National Health Systems* [14]. This monograph, sponsored by the Global Health Workforce Alliance (GHWA) and WHO, was a prominent advance for the global CHW movement in the sense that it had the imprimatur of WHO. It also strongly advocated for the inclusion of CHW programmes as an integral part of national health systems and inclusion of CHW programming as part of the country’s strategic planning for HRH [14]. The report called on countries to establish a national plan of action finalized by a working group of stakeholders to develop and improve CHW programmes that are integrated into the health system [14].

While CHW programmes of different sizes and shapes exist today in most countries, answers to questions of how they should be developed, expanded, and strengthened are very diverse and often remain unresolved. Kok and colleagues in 2017 presented a complex set of factors that define the hardware and software of health systems and interventions that influence CHW performance in a certain context—where hardware comprises supervision, training, incentives, communication, and supplies, and software comprises ideas, interests, power, values, and norms of the health system [15]. A systematic review by Scott and colleagues in 2018 synthesized the findings from 83 reviews on CHW programmes from LMICs to understand their design and operations in the health system. This review found that CHW programmes that (1) encourage community ownership, (2) provide supportive supervision, ongoing education, and materials and supplies for undertaking tasks, and (3) integrate the programme into the existing health system are able to strengthen the sustainability and foster the credibility of the CHW programme [16].

The 2019 resolution of the World Health Assembly [17] notes with concern the “uneven integration” of CHWs into health systems. This “uneven integration” is partly a result of the lack of integration of CHW programmes into planning, coordination, and partnerships in the health system more broadly.

An analytical assessment that we report elsewhere in Paper 10 of this series [18] attempts to unravel the complex and interconnected factors that influence the performance of CHW programmes in LMICs. The 2018 *WHO Guideline on Health Policy and System Support to Optimize Community Health Worker Programmes* [19] does address many of these factors, including programme design, policy coherence, health systems support, and financing. However, none of their 15 areas of recommendations refer specifically to planning, coordination, or partnerships. However, as we describe in the Discussion section, the guideline does, at the very end of the document, provide a set of general implementation considerations that are directly relevant to planning, coordination, and partnership formation. We review these in the Discussion section.

This article addresses questions, challenges, and strategies for stakeholders to consider while planning the introduction, expansion, or strengthening of a large-scale CHW programme and the complex array of coordination and partnerships that need to be considered as CHW programmes cross the threshold into a new era in which their importance for the health system and improvement of population health are more fully appreciated.

## Methods

Over the past decade, the authors have personally engaged in key consultations with various government leaders and experts from across policy, implementation, research, and development organizations. These include global consultations on CHWs and global forums on HRH between 2010 and 2014 (Montreux, Bangkok, Recife, Washington DC). They also build on the authors’ direct involvement with the GHWA. We have also carried out a limited, non-comprehensive review of the literature on this topic. Finally, we draw on the recently released compendium of 29 case studies of national CHW programmes from 27 countries [12] and informal consultation with key informants identified by the authors. The article also draws on the 2014 chapter by the authors on this topic in the book, *Developing and Strengthening Community Health Worker Programs at Scale: A Reference Guide and Case Studies for Program Managers and Policymakers* [10].

**Table:** 

**Key message box 2**
The lack of integration and synchronization of CHW programmes with local health systems and local health needs often led to gaps and fragmentation in service delivery

## Results

### Why are planning, partnerships, and coordination needed for CHW initiatives?

Historically, in many countries, CHW programmes have emerged piecemeal and were often centred on individual projects—frequently, vertical programmes with separate funding mechanisms from donors. The lack of integration and synchronization with local health systems and local health needs often led to gaps and fragmentation in service delivery. For instance, some areas might be served by more than one programme, while others had none. Concomitantly, CHW programmes have often emerged without careful planning, without well-developed partnerships and coordination, and without a well-defined and thoughtful plan for long-term financing and sustainability.

The multisectoral dimension of HRH more broadly is at the core of the global health workforce agenda [20]. Many complex and diverse HRH challenges such as the education of the health workforce, workforce regulation and accreditation, labour market and employment practices, in- and out-migration, regulations, and quality assurance are invariably beyond the power of a single actor to duly address. They require multilevel coordination and collaboration among different players and actors. Similarly, CHWs, like other health workforce cadres, have multisectoral dimensions and implications.

The planning, financing, management, implementation, and monitoring of CHW initiatives require synergized actions from and interaction among various sectors and stakeholders, including different government agencies and departments such as the ministry of health (MOH), other government ministries (education, labour, finance, and local government), regional and local governments and municipalities, regulatory bodies, professional associations, the private sector, civil society organizations, nongovernmental organizations (NGOs), and local communities, not to mention international development partners including bilateral donors and United Nations (UN) agencies. Therefore, coordination and synchronization among the various sectors and stakeholders are vital, particularly for policy development, planning, implementation, management, monitoring, and evaluation. The importance of these in the successful implementation of various CHW programmes has been underscored in the recent book on 29 national CHW programmes [12]. To do this effectively, a robust coordination process and a suitable mechanism is required that can bring these stakeholders together on a common platform and agenda to ensure harmonized actions.

There are two well-documented examples in which planning, partnerships, and coordination (or lack thereof) were particularly important to the success of a national CHW programme: the National Village Health Guides programme in India and the Health Extension Programme in Ethiopia. As mentioned earlier, the Village Health Guides programme was hastily planned in 1977, with minimal coordination and minimal engagement of stakeholders (including the lower levels of the government health system at the district level), as an attempt to win votes for an impending national election, leading to a lack of strong sustained political and financial support over time and the eventual demise of the programme [21]. The Health Extension Programme, on the other hand, was carefully planned in the early 1990s in coordination with stakeholders and with strong and consistent political leadership over time from the Prime Minister and Minister of Health (who is currently WHO Director-General, Dr Tedros Ghebreyesus Adhanom), leading to what has become a model CHW programme for Africa [22–24]. We elaborate on these and other examples in the Discussion section.

### What are the challenges of planning, coordination, and establishment of partnerships for CHW initiatives?

CHW initiatives face complex and critical challenges that are mainly multidimensional and multisectoral. Health systems in lower-income countries are generally weak and do not have the capacity to adequately support the delivery of essential health services to the target population. This also constrains the capacity of health workers to operate effectively in these settings.

There is no single typology for CHWs internationally or within countries (see Paper 5 in this series on roles and tasks of CHWs [25]**)**. Rather, there exists a broad array of types of CHWs with a diverse set of labels and categories describing them and with widely different training, assigned and unassigned tasks, and management and support systems.

There is usually not a nationally recognized and integrated system of training of CHWs leading to a nationally recognized certificate. In-service and continuous training programmes are usually insufficient and sporadic, are often funded by external donors, and are frequently provided by NGOs with short-term funding. There are diverse models of career development and incentive structures. These issues are addressed in Papers 6 (on training) and 8 (on incentives and reimbursement) [26, 27].

There are many additional challenges. A strong political commitment to CHW initiatives is often lacking even in the face of profound shortages of health workers, especially in rural areas. Customarily, CHW initiatives are seen as under the purview of the MOH; therefore, other sectors and partners, including NGOs who may have their own CHWs, may not become meaningful stakeholders.

Policies and plans for CHW initiatives are frequently deficient, particularly with respect to training, certification, management and supervision, and funding. This—combined with limitations of health system capacity, political instability, transparency issues, and competing priorities in the MOH and government—often leads to a highly suboptimal rollout and operation of the CHW programming.

Financial resources to support CHW initiative are usually insufficient, and sustainability is not guaranteed, as discussed in Paper 4 in this series on financing of CHW programmes [28]. The lack of engagement with stakeholders leads to a vast untapped potential and loss of opportunities for public–private partnerships. There is a lack of effective coordination mechanisms and harmonization of actions, leading to fragmentation of the CHW initiative. This is coupled with weak linkages to existing national policy development and health-related coordination mechanisms.

**Table:** 

**Key message box 3**
The lack of effective coordination mechanisms and harmonization of actions across diverse stakeholders in different sectors leads to fragmentation of the CHW initiatives in the context of weak linkages to national health policy development

### What are the policy options for planning, coordination, and establishment of partnerships for CHW initiatives?

**Fig. 1:**
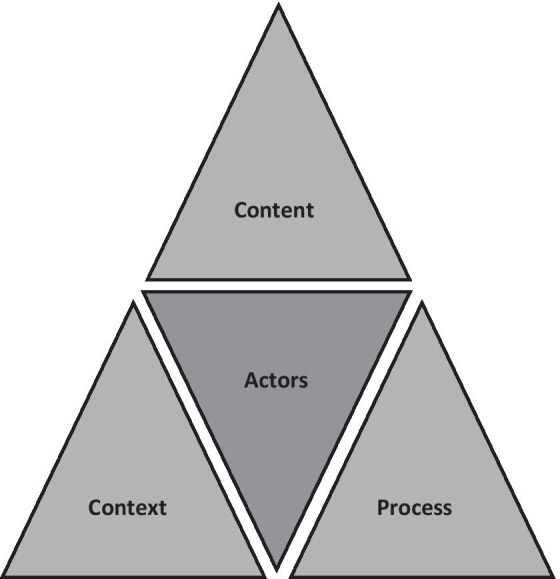
The health policy triangle (adapted from Walt and Gilson [29])

The Walt and Gilson Health Policy Framework (Fig. 1) is a guiding source to systematically formulate a policy triangle around CHW programme planning, considering different factors that may play a role such as the actors, context, process, and content [29]. The framework mostly helps in understanding the policy-making process; however, its components allow for exploration of *actors* that are involved, the *content* of actions, the *context* in which they are being applied, and the structures of the *process*.

The complexities underpinning CHW initiatives call for multisectoral policy options that require careful attention to every critical step, including planning, development, recruitment, retention, management and supervision, and career development. Multisectoral oversight, political commitment, and coordinated actions by the government and related stakeholders are also fundamental. Conjointly developed solutions endorsed and supported by the government have a better chance of acceptance by different constituencies and stakeholders. Such solutions entail planning, coordination, and establishment of partnerships among various stakeholders and partners from different constituencies and sectors to develop shared policies and plans in addressing the health challenges that CHWs are helping to mitigate. These should be duly incorporated into the overall HRH system and national health agenda.

Creating an environment that is conducive for multisectoral coordination and synchronizing actions requires inclusive engagement and wider consultation with relevant stakeholders. In addition to the MOH, these may include other public sector institutions and agencies, academics and researchers, professional forums, staff associations, regulatory and governing bodies, civil society, NGOs, and the private sector. Along with that, productive partnerships with local communities in shaping the CHW agenda is of paramount importance, particularly in identifying local health needs, setting priorities for CHW roles and responsibilities, identifying candidates for recruitment as CHWs, providing local support, and engaging in CHWs’ supervision and performance evaluation.

In this context, the establishment of an effective coordination process should offer a workable platform that can engage all partners and stakeholders—benefiting from their knowledge, competencies, and resources—to yield tangible results. National coordination processes for the entire health workforce including CHWs should be institutionalized and bring a suitable national perspective to the development of policies and plans. This increases ownership and augments the likelihood of more sustainable solutions with mutual accountability of stakeholders. This platform facilitates policy dialogue among the stakeholders to reach a consensus on policies and priorities.

In many countries, the MOH, being the principal stakeholder, is in a lead position and provides stewardship to the coordination process, convening and aligning related sectors by bringing them on board during the key phases of planning, resource mobilization, programme implementation, and monitoring of the programme’s progress and effectiveness. This requires the sharing of information and insights through formal communications channels. This coordination platform enables channelling and streamlining support to other sectors and stakeholders, enabling them to more effectively perform their roles for promoting the CHW agenda. This can be through their formal orientation and awareness raising as well as through building their capacities in policy development, planning, implementation, monitoring, and providing for accountability. A coordination platform can also harmonize the efforts of development partners and UN agencies with national goals, priorities, and plans. This same coordination platform can address the financial and technical support needs required for implementing effective CHW programmes.

### What are approaches to national-level multi-stakeholder coordination?

To promote a multisectoral process and a coordination mechanism to enable related sectors to work together with harmonized and synchronized efforts, there are several national multi-partner coordination mechanisms. These include the sector-wide approaches (SWAps) [30] and joint government and donor review processes, country coordinating mechanisms (CCMs) [31] introduced by the Global Fund to Fight AIDS, Tuberculosis, and Malaria, the International Health Partnership (now UHC2030) [32], and development of global compacts, national HRH observatories, and Country Coordination and Facilitation (CCF) approaches introduced by the GHWA [33]. There is no one-size-fits-all coordination, and it is critical to adapt and use the coordination process that best suits the country’s situation and needs.

SWAps call for a partnership in which the government and development agencies (under government leadership) interact together in the formulation of policy [34]. Under the SWAp, project funds contribute directly to a sector-specific *umbrella* and are tied to a defined sector policy under government authority.

The CCM is central to the commitment of the Global Fund to Fight AIDS, Tuberculosis, and Malaria to local ownership and participatory decision-making. CCMs involve representatives from both the public and private sectors, including governments, multilateral or bilateral agencies, NGOs, academic institutions, private businesses, and people living with the targeted diseases. These country-level multi-stakeholder partnerships develop and submit grant proposals to the Global Fund based on priority needs at the national level. After grant approval, they oversee progress during implementation. For each grant, the CCM nominates one or more public or private organizations to serve as the principal recipient that receives and distributes the funds [31].

The International Health Partnership, now called UHC2030 [32], is a group of partners committed to improving health in LMICs, including advancing the CHW agenda. Partners include international organizations, bilateral agencies, and country governments. They all signed the UHC2030 Global Compact for achieving the health-related SDGs. UHC2030 achieves results by mobilizing national governments, development agencies, civil society, and others to support a single, country-led national health strategy, a single monitoring and evaluation framework, and a strong emphasis on accountability [32].

The National HRH Observatory is a WHO-supported initiative established by the MOH as a national resource for producing, sharing, and utilizing health workforce information and evidence to support HRH policy implementation. It involves a network of all information resources and stakeholders for health workforce development in the country. The network monitors and documents the implementation of HRH policy and strategies to harmonize the mobilization of resources, strengthen HRH managerial structures, establish training institutions, and scale up the training of CHWs [35].

The CCF approach, conceived by the GHWA in 2009, focuses on establishing and/or strengthening multi-stakeholder coordination processes around the HRH agenda [33]. The CCF approach brings together key stakeholder constituencies to agree on priorities and then to develop and fund a single national plan to address HRH challenges.

Implementing the CCF approach involves an analysis of stakeholder capacity; establishment of HRH committees and technical working groups for developing evidence-based, comprehensive, and costed HRH plans; and engagement of stakeholders in resource mobilization, implementation, and monitoring and evaluation of the approach’s implementation. This approach relies on principles such as (1) the use of existing coordination mechanisms when possible; (2) inclusive representation of HRH stakeholder constituencies; (3) coordinated leadership and stewardship; (4) defined roles for stakeholders; (5) coherent strategies linked with national health policies; (6) joint efforts and actions arising from increased investments in HRH; and (7) linkages with other coordination mechanisms.

The key intent of the CCF mechanism is to put the MOH and the government in the lead position, institutionalizing coordination within existing systems and structures instead of developing new structures. There are diverse challenges in establishing the CCF framework in different settings. The most common challenges include inadequate motivation and commitment by the national authorities, diverse CHW models, limited awareness in related sectors on their role, and inherent difficulties of coordination over the long term (as opposed to the ease of coordination in the short term to achieve a specific goal). There are a number of examples where countries leveraged their existing institutions and built a coalition around them for the benefit of their CHW programmes. Examples include Afghanistan, Cameron, Indonesia, Sudan, Zambia and Zimbabwe [36]. Among these, we discuss Afghanistan and Indonesia further in the Discussion section.

**Table:** 

**Key message box 4**
Effective co-ordination of CHW programmes relies on principles such as (1) use of existing coordination mechanisms when possible; (2) inclusive representation of HRH stakeholder constituencies; (3) coordinated leadership and stewardship; (4) defined roles for stakeholders; (5) coherent strategies linked with national health policies; (6) joint efforts and actions arising from increased investments in HRH; and (7) linkages with other coordination mechanisms

With this paradigm, the CCF enables governments to take the necessary leadership in the planning, coordination, implementation, and management of HRH development at the country level and to work with partners aligned to support this priority pillar of the health system.

Under mounting pressure from its constituencies, the GHWA developed the CCF framework that provided advice and guidance on intersectoral and multi-constituency collaboration to accelerate the implementation of a country’s HRH agenda. As a result, the CCF approach was implemented in several countries, demonstrating positive outcomes in the GHWA’s external and internal evaluations. The GHWA completed its 10-year lifespan as a partnership of the WHO in 2016, replaced by the Global Health Workforce Network. At this critical point, the GHWA legacy report reviewed and illustrated its policies, initiatives, and their impacts [37]. The CCF framework for national planning and development was extensively discussed in the report. The report recognizes its systems perspective and the GHWA Board’s focus on the critical gap in HRH planning coordination at national levels.

The legacy report notes that “several key informants regarded GHWA’s CCF work as a major contribution, most noting that where it was applied, the CCF process brought together diverse participants within each country, often including hard-to-reach political leaders” [37]. The legacy report also indicated that “[i]mplementation issues notwithstanding, the CCF Framework is currently viewed by many key informants as a valuable methodology for HRH improvement and amongst GHWA’s most significant achievements”.

### How can initiatives for CHWs and other FLHWs be best coordinated?

Although some countries have been able to implement CHW programmes within their national health systems by exercising national leadership, still a number of countries require support from donors, international development partners, and external technical advisors. Many partners are engaged in supporting CHW programmes in various countries but find fragmentation of policies and programmes to be a big challenge. This calls for harmonized and synchronized actions that support national needs. Particularly, to deliver on UHC at the country level by 2030, the global health community needs to support the country to address critical gaps and inefficiencies at all levels.

In 2012, four separate global consultations^1^ highlighted the significance of CHWs and other FLHWs in achieving health goals in LMICs. The GHWA in consultation with key partners developed a synthesis paper [38] derived from the outcomes of these consultations. The key messages for common actions were formulated for the domains shown in Table 1.

**Table 1:** Priorities for planning, coordination, and partnerships for CHW programmes arising from global consultations in 2012

Broader recognition of the importance of a stronger role for CHWs and their integration into health systems
National-level consultations and advocacy
National-level multi-stakeholder collaborations
Alignment and synergies among stakeholders and partners
Narrowing of the gap between current evidence and practice
Stronger monitoring and assessment of CHW programmes
Stronger shared accountability

Moving onward, the key partners of the GHWA jointly developed three working papers that together have become a framework for harmonized partners’ actions, also known as the CHW Framework for Partner Action, which has proven quite useful in practice in a number of countries. The papers that make up the CHW Framework include:

- *A Framework for Partners’ Harmonized Support: Community Health Workers and Universal Health Coverage* [39]
- *Monitoring and Accountability Platform: For National Governments and Global Partners in Developing, Implementing, and Managing CHW Programs* [40]
- *Knowledge Gaps and a Need-Based Global Research Agenda by 2015: Community Health Workers and Universal Health Coverage* [41]

These papers propose a set of guiding principles to support countries and their partners in their efforts to harmonize donor support in accordance with commitments by all partners to collaborate at the global and national levels. They seek to build greater synergies across CHW programmes—within countries and between countries—guided by the national leadership, national strategies, and nationally agreed systems for monitoring and evaluation. Part of the overall goal is to improve efforts to integrate CHWs into the broader health system, with a particular focus on effective linkages between community-based and facility-based health workers at the front lines of service delivery, so that all persons and communities receive the health services they need.

This CHW Framework for Partner Action is structured around a “Three-Ones” approach,^2^ with three overriding principles for harmonization in line with the Paris Declaration on Aid Effectiveness [39] as follows:

- One national strategy as the shared basis for CHW programme investment and alignment of all partners
- One authority respected by all partners and clearly identified at the national level that also has delegated its authority to an appropriate entity at the district level
- One monitoring and accountability framework as the basis for reporting and accountability to all partners

Convened by the GHWA and other key partners, a global consultation was held during a side session at the Third Global Forum on Human Resources for Health at Recife, Brazil, in 2013. The group endorsed the CHW Framework for Partner Action and concurred on a joint commitment [42] to work together to adapt, apply, and implement the CHW Framework, fostering harmonization and synergies, accountability, and joint action on critical knowledge gaps, and reaching out to all stakeholders engaged with CHW programmes. The commitment promotes alignments with and implementation by partners working toward scaling up CHW programmes through their efforts at global, regional, and national levels.

This 2013 meeting in Brazil and the 67th World Health Assembly held in Geneva in 2014 were global forums to discuss the Global Strategy on Human Resources for Health 2030. Subsequently, the 69th World Health Assembly in May 2016 adopted the resolution of this Global Strategy. This resolution embraces key actions related to the health workforce and their implication in health policy and programmes. The most pertinent one for our purposes here (related to planning, coordination, and partnerships) states the following: “Development partners, including bilateral partners and multilateral aid mechanisms, will augment, coordinate and align their investments in education, employment, health, gender and labour in support of domestic financing aimed at addressing national health workforce priorities.”

*The Global Strategy on Human Resources for Health: Workforce 2030* [20] is primarily aimed at planners and policymakers of Member States, and the relevant stakeholders in the health workforce area, including public and private sector employers, professional associations, education and training institutions, labour unions, bilateral and multilateral development partners, international organizations, and civil society. The strategy calls for increases in health financing for the recruitment, development, training, and retention of the health workforce by 2030.

In this context, bilateral and multilateral agencies are urged to increase synergies in official development assistance towards education, employment, gender, and health in support of national health employment and economic growth priorities. This also includes aligning the investment in HRH for health to address the current and future needs of the population and health systems, considering labour market dynamics and education policies.

Stakeholders and international partners are encouraged to recognize the investment in the health workforce as productive investments not only for its health benefits but for socioeconomic development as well. Development partners are expected to align their investments for HRH with coordinated, long-term national needs as expressed in national plans for the health sector.

UN Secretary-General Ban Ki-moon established the High-Level Commission on Health Employment and Economic Growth in March 2016 [43]. The Commission, after rigorous review, consultation, and deliberation facilitated by the International Labour Organization, the Organisation for Economic Co-operation and Development, and WHO, made a set of 10 recommendations to stimulate and guide the creation of at least 40 million new jobs in the health and social sectors, and to reduce the projected shortfall of 18 million health workers, primarily in LMICs, to implement the SDGs by 2030 [43].

In essence, these recommendations call for intersectoral collaboration and multi-stakeholder cooperation. Its recommendations pertaining to “Partnership and Cooperation for HRH” calls for promoting intersectoral collaboration at national, regional, and international levels; engaging civil society, unions and other health workers’ organizations, and the private sector; and aligning international cooperation to support investments in the health workforce as part of national health and education strategies and plans. Toward that, actions are required across all sectors involved with the health labour market. These intersectoral processes, with well-defined accountability mechanisms, predominantly engage public and private sectors, civil society, trade unions, health worker associations, NGOs, regulatory bodies, academic institutions, and development partners to help in operationalizing the SDG 2030 vision.

Subsequently, the Fourth Global Forum on Human Resources for Health, November 2017, held Dublin, Ireland, focused on “Building the Health Workforce of the Future” [44]. Stakeholders across sectors, regions, and nations were invited to join in supporting the implementation of the Global Strategy and the High-level Commission recommendations. The Dublin Declaration called upon the relevant stakeholders to strengthen their collaboration to expand and transform investments in the health and social workforce, and to align social accountability, health workforce education, skills, and employment to address priority population needs.

## Discussion

**Table:** 

**Key message box 5**
Since the mid-2010s, the policy discourse on CHWs has merged with that for PHC more broadly as a key strategy for achieving the global health goals for 2030 of UHC, ending preventable child and maternal deaths, and the SDGs. Moving from a focus on vertical, disease-oriented programming to an integrated PHC approach that has a strong CHW component is occurring, but only slowly, unfortunately. Bringing CHWs into the formal HRH framework for planning, coordination, and partnership formation will require a paradigm shift in enhancing their recognition and status in the health system

The array of challenges confronting planning, coordination, and partnership formation for establishing, expanding, and strengthening CHW initiatives is daunting, to be sure. And the processes put forward for carrying these out are complex and unwieldy. One could easily adopt the position that such a task is impossible. No doubt, the slow progress made in incorporating CHWs into the health systems of many countries is a reflection of this reality.

However, there are notable examples in which these obstacles have been overcome, and the benefits for the health of the population—and especially for mothers and children—have been noteworthy [12]. The pendulum has shifted back to a recognition that CHWs are essential for achieving the SDGs, UHC, and ending preventable child and maternal deaths [45]. So the question changes from *whether* to *how best* to create strong CHW programmes. There is no “cookie-cutter” solution, and each country has to find its own unique way to do this.

### Recent trends in policy discourse regarding the planning, coordination, and partnership formation for CHW programmes

Since the mid-2010s, policy discourse that concerns CHWs has shifted away from CHWs specifically toward the broader scope of PHC (including the Astana Declaration [46] and the PHC Operational Framework [47] launched by the new WHO special programme), UHC, and the critical contributions that CHWs will make to ending preventable child and maternal deaths and achieving the SDGs by 2030 [48].

The Community Health Roadmap [49] is an innovative collaboration between traditional multilateral and bilateral donors, private funders, and global health leaders including USAID, the World Bank, WHO, the Bill & Melinda Gates Foundation, The Rockefeller Foundation, United Nations Children’s Fund (UNICEF), and Office of the WHO Ambassador for Global Strategy established in 2019 to better align existing resources and to attract new resources to community health and support countries in achieving their goals for PHC, UHC, and SDG3. The Roadmap aims to promote and coordinate investments in community health globally as well as nationally by elevating national priorities for community health through donor collaboration, global advocacy, and cross-country learning [50].

At the national level, there is a growing push for greater coordination with education ministries for creating a pathway for training and advancement of the CHW workforce. The formation of working groups is also underway within various ministries of individual countries to digitize and strengthen the data collected by CHWs. Having said that, however, it is still unfortunately the case that fragmentation and vertical, disease-specific programmes remain dominant in many countries, with the result being that the planning, coordination, and partnership establishment for CHWs remains fragmented and top-down in orientation.

In the opinion of some, the coordination mechanisms described previously have been forced on countries in the sense that they are required to have them in order to receive funding, and there are different coordinating mechanisms for different kinds of initiatives. Therefore, their effectiveness has been limited. Furthermore, CHWs have not been a prominent component of the activities being coordinated. The Global Fund has been supportive of including CHWs in the proposals from the CCMs, though they are mostly for top-down, disease-specific initiatives (HIV, tuberculosis [TB], or malaria).

### Countries that have a strong record in planning, coordination, and partnership development for CHW programming

National planning, coordination, and partnership development have been prominent for a number of countries with strong CHW programmes. Among these are Afghanistan, Brazil, Ethiopia, India, Indonesia, Liberia, and Nepal. Coordination with donors has been a particularly important element of this process in all of these countries except for Brazil and India. Ethiopia is considered to have been effective in planning for a national CHW programme with strong central and national leadership that at the same time drew in support from donors and led to better coordination among partners.

#### Afghanistan

Afghanistan's CHW programme is a model of planning, coordination, and partnerships [51]. Beginning in 2003, after the removal of the Taliban, donors working with the government planned together, coordinated their efforts, and forged partnerships with NGOs to create a strong programme that brings together what had previously been a plethora of independent NGO CHW programmes. The government contracts out the provision of PHC services to NGOs at the district level, and these NGOs train and supervise CHWs.

#### Brazil

Brazil is one of four countries selected by the Exemplars in Global Health Partnership because of the demonstrated exemplary performance of its CHW programmes, along with Bangladesh, Ethiopia, and Liberia [52, 53]. Brazil’s CHW programme has been evolving since the early 1980s when it was first introduced as an emergency pilot project in one of the poorest areas of Brazil (Ceará) in the midst of a serious drought. Because of its demonstrated local effectiveness, the CHW programme gradually scaled up through broad civic participation with the government as the country expanded and improved its PHC system, within which the CHW programme is an integral part. What gradually evolved was a national PHC programme that had strong participation of local municipalities in funding and governance but within a national framework of standards and funding. Brazil’s progress in improving the health of its population as a result of its strong community-based PHC system has also been exemplary.

#### Ethiopia

There is general agreement that Ethiopia is one of the best examples of a country-led vision for a national CHW programme in which national leadership has enabled strong planning and multisectoral coordination that included external donors. In 2008, Ethiopia was one of the first developing-country signatories to the International Health Partnership Plus (IHP+) Compact, which intended to align donor countries and aid recipient nations to accelerate progress towards MDGs through more effective administration of foreign aid. In Ethiopia, the health sector concept of “One Plan, One Budget, and One Report” fit well with the principles that were reflected in the IHP+, and Ethiopia’s newly created CHW cadre of health extension workers (HEWs) was a central part of its health sector transformation.

Through an annual review meeting process, development partners were granted the opportunity to assess and influence the national health policy process [54]. Of the 10 developing country governments first participating in the IHP+, Ethiopia was one of the stronger performers [55]. The Federal MOH’s and development partners’ commitment to country-led planning and joint budgeting and monitoring facilitated rapid progress in the strengthening of Ethiopia’s PHC platform. Through strong government-led planning, coordination, and establishment of partnerships, a flexible approach was instituted that allowed for iterative adjustments as the programme was scaled up nationally [56, 57].

The government and its leaders were effective in utilizing vertical disease-control funding to build an integrated PHC platform in which HEWs provided services that are normally part of vertically funded parallel programmes (e.g., immunizations, family planning, and HIV-related activities) while at the same time providing broader maternal-child health and acute-care services [58]. This demonstrated leadership helped convince many donors to provide financing for the PHC system (and funding for HEWs as a provider of integrated services) rather than following the more common practice of supporting separate disease-specific or project-specific activities.

Ethiopia’s CHW programme was the subject of an in-depth review by the Exemplars in Global Health programme [52]. Features that contributed to the success of this CHW programme were (1) political commitment at the highest level (of the prime minister) and across government, (2) embedding the CHW programme in the PHC system, (3) attracting external resources and managing donors effectively, channelling their investments and expertise to support the country’s flagship programme, (4) development of a pilot project that could be adapted prior to national scale-up, (5) engagement of the Federal MOH with the Ministries of Education, Finance and Economic Development, Public Works, and Urban Development to help roll out and expand the programme. The Ministry of Education provided technical and vocational educational training and resources, including teachers, to train the HEWs. The Ministry of Public Works and Urban Development’s subnational offices facilitated the construction of health centres and health posts by providing contractors and supervision and ensuring the quality of the construction. The Ministry of Finance and Economic Development engaged the HEWs as civil servants and paid their salaries from block grants transferred to the regions and woredas, (6) subnational ownership and responsibility, and (7) ongoing adaptation and upgrading through annual meetings that included the MOH and partners [52].

#### India

Incorporating lessons from the failed national Village Health Guides programme (mentioned previously and discussed further below), India developed a strong national CHW cadre of accredited social health activist (AHSA) workers, which is now more than 1 million strong [59]. An exemplary process was developed for routine planning and coordination during biannual reviews to provide the opportunity to check on the status of the programme, troubleshoot, and respond to identified challenges. India has a semi-independent entity, the National Health Systems Resource Centre [60], that performs an important leadership role for planning, coordination, and facilitation of partnerships for the AHSA programme, including its integration into the PHC system.

#### Indonesia

Indonesia’s system of CHWs (*kaders*) arose from within an existing set of women’s organizations of family welfare clubs under the leadership of a separate government ministry—the Department of Home Affairs. The initial focus was on self-help activities and family planning, and then it expanded to growth monitoring and nutrition advice, provision of oral rehydration salts for childhood diarrhoea, vitamin A supplementation, and immunization at the time of monthly meetings in the village (called *posyandus*, run by *kaders*) [61]. The evolution of this programme over almost five decades has involved multisectoral planning, coordination, and partnerships of the Ministry of Home Affairs with the MOH and the National Population and Family Planning Board, with strong support from then-president Suharto.

#### Liberia

Liberia is another one of four countries selected by the Exemplars in Global Health Partnership because of the demonstrated exemplary performance of its CHW programmes, along with Bangladesh, Brazil, and Ethiopia [52, 53]. Following a protracted civil war that ended in 1996 and the devastating Ebola outbreak in 2004–2015, the government of Liberia together with donor partners and civil society joined together to upgrade its poorly functioning and fragmented programme of volunteer CHWs by updating its National Community Health Services Policy and Strategic Plan, leading to the formation of a paid CHW cadre of community health assistants in communities more than 5 km from a health facility. As a result of a careful, participatory, and well-coordinated planning process that included many internal and external stakeholders, a highly effective national programme has now been implemented that has achieved impressive results in expanding basic health services and improving the health of the population [62, 63].

#### Nepal

Nepal had more than two decades (from the 1990s to the 2010s) of close planning, coordination, and partnerships between the government with UNICEF and with USAID-funded partners to support the development of female community health volunteers (FCHVs), helping to make this an exemplary CHW programme. Several national CHW programmes in Nepal preceded this one, and these were not successful. FCHVs, many of whom had worked in one of the previous CHW programmes, first began with vitamin A distribution and then gradually took on new tasks, including community case management of childhood pneumonia. The National Organization of Women and external donors were key partners with the MOH in their work.

### Examples of countries that have faced challenges in planning, coordination, and partnership formation

#### Bangladesh

Bangladesh is another one of the four countries selected by the Exemplars in Global Health Partnership because of the demonstrated exemplary performance of its CHW programmes, along with Brazil, Ethiopia, and Liberia [52, 53]. Bangladesh has been a pioneer in the use of CHWs and in health improvements to which CHWs have made important contributions. However, in terms of planning, coordination, and partnership formation, there have been ongoing challenges.

For almost a half-century now, Bangladesh has had two separate cadres of CHWs in the public sector, the family welfare assistant (FWA) cadre under the auspices of the Directorate of Family Planning of the Ministry of Health and Family Welfare, and the health assistant (HA) under the auspices of Directorate of Health Services [64]. These two CHW cadres complement each other at the local level in the sense that their formal roles and responsibilities do not overlap, but historically, there has been a lack of close coordination of the work of the two cadres. Tensions between the two cadres became quite prominent recently when the Directorate of Health Services placed HAs into a higher pay grade without a similar move by the Directorate of Family Planning for the FWAs even though they have comparable pre-service qualifications and job responsibilities.

And, in addition, there are a large number of CHWs working with NGOs in Bangladesh, most notably the two levels of CHWs (*shasthya shebikas* and *shasthya korbis*) who work with Building Resources Across Communities (BRAC), which is national in scope [65]. Again, the degree of coordination at the local level between NGO and government CHWs has been limited because each group of CHWs has separate supervisory systems. More recently, government of Bangladesh has introduced yet another CHW cadre, community healthcare providers (CHCPs), to provide frontline health services at community clinics [64].

Although there have been long-standing problems of planning, coordination, and partnership formation at all levels for CHW functioning, there is emerging an interesting and potentially effective approach to micro-level planning, coordination, and partnership formation in which community representatives and local government leaders are now guiding local activities of CHWs to harmonize their work and enhance its effectiveness [66].

An assessment of the SWAp from 1998 to 2013 in Bangladesh [67] found progress in planning, coordination, and partnership formation in the overall health sector, but made no mention of planning, coordination, and partnership formation for CHWs specifically, although a revitalization of community clinics was mentioned. Despite these issues, Bangladesh has been a leader in implementing strong CHW programmes [52, 68]. Even so, Bangladesh faces challenges to planning, coordination, and partnership formation that are common in many countries, as described in the Exemplars’ report.

Often, countries have a patchwork of CHW programmes managed by various NGOs, each focused on its own narrow list of health challenges, and with different remuneration, training, and performance standards. Such inefficient fragmentation scatters limited resources and creates both redundancies and gaps in PHC. Additionally, it poses challenges for government ownership and programme management. Moreover, the differences in CHW skills, services, and training between the various CHW cohorts working in a single community can be confusing to patients, and decrease their trust, leading to less demand for services. In addition, fragmentation undermines the critical connection between CHWs and facility-based PHC systems. Data in such fragmented systems are generally not shared across the health system and therefore does not contribute to building robust and effective health management information systems [52].

#### Cambodia

Cambodia’s CHW programme has had weak government leadership, and its CHWs have not been well integrated into the government health system and have been excluded from the health sector planning process [69]. This has led to a lack of resources for the programme, lack of support from the government at the national and local levels, and a fragmented programme that is largely influenced by external aid objectives and vertical programmes [69].

#### India

We have mentioned previously the failed national Village Health Guides programme that operated from 1997 to 2002 [13, 70]. In this case, donors were not involved. There was a lack of careful planning, coordination, and partnership-building within the MOH (particularly at the lower levels). Fortunately, the lessons learned from this were put to good use, as the ASHA CHW programme emerged later in response to the need for a stronger CHW presence in India, as we discuss above.

#### Madagascar

Madagascar has had the unfortunate circumstance of weak central leadership for its CHW programme, with a bifurcated programme: one cadre of CHWs in the MOH and another in the nutrition sector. Furthermore, donor partners adapted these CHWs to fit their own paradigms in subnational sections of the country, leading to a lack of uniformity in standards, policies, roles, and functions as well as minimal coordination among partners [71]. Efforts to unify this system are now underway, fortunately.

#### Myanmar

As a result of weak central government commitment to a strong CHW programme and weak government leadership, Myanmar’s CHW programme remains a fragmented one driven by vertically funded, disease-specific programmes [72]. There are seven different CHW cadres in Myanmar: auxiliary midwives, CHWs, malaria volunteers, TB volunteers, village health workers, maternal health workers, and trained traditional birth attendants. Each cadre functions relatively independently and is guided by different sections in the MOH. Planning, coordination, and partnership formation are limited because each section in the MOH has its own independent donor funding, and the directors of these sections are reluctant to sacrifice control over and funding for their own programme.

#### Uganda

Two recent publications point to the complexities of planning, coordination, and partnership formation that occur in the process of introducing a new cadre of paid, professionalized CHWs modelled after Ethiopia’s HEWs to complement the existing volunteer village health teams [73–75]. The issues raised provide an important lesson in the need for participatory planning, coordination, and partnership formation at all levels of the health system down to the community level.

### Advice from the 2018 WHO guideline

The WHO guideline on CHWs released in 2018 [19, 76] is notably silent in making formal recommendations on the issues of planning, coordination, and partnership formation. But it provides a final section following the formal specific recommendations entitled “General Implementation Considerations” that highlights some important principles that should be considered as part of the process for planning, coordination, and partnership formation for CHW programmes. Here, we summarize some of these principles.

#### Plan for the long term

Planning, coordination, and partnership formation should be carried out with a long-term perspective and with dedicated long-term financing. As the guideline states, “attempting to set up and run a large-scale CHW initiative on a shoestring budget is likely to yield disappointing results” [19] (p. 72).

#### Foster political will

The key determinant of success will be sufficient political will to make it possible to build a CHW programme that can achieve its full potential [19].

#### Take into account the perspective and interests of CHWs

The WHO guideline stresses the importance of involving CHWs in the policy dialogue, respecting their labour rights and their need for safe and decent working conditions, and protecting them from discrimination, coercion, and violence [19].

#### Take a “whole of system” approach

CHW programmes cannot be initiated, expanded, or strengthened in isolation without taking into account the population health needs, the health system’s current strengths and weaknesses, and the role of CHWs vis-à-vis other health workers. CHW programmes must be integrated into the health system rather than implemented as a stand-alone entity. Therefore, countries should plan their health workforce as a whole rather than planning for a single occupational group, which risks “fragmentation, inefficiency and political inconsistency” [19] (p. 71). The obvious danger here, however, is that those guiding this holistic approach may not value CHWs or see their contribution as central and integral to optimizing health system performance.

#### Consider the capacity of the health system to support a strong CHW programme

The WHO guideline rightly emphasizes the need to consider (1) the capacity of the health system to provide appropriate support to CHWs and (2) the needed reforms in the health system to provide the needed support. The supporting functions that the guideline mentioned were training and supervision of CHWs, provision of competency-based certification, effective management of the CHW programme, protection of CHWs from malpractice risks, remuneration in a timely and adequate manner, creation of appropriate channels for linkages and referrals, and procurement of commodities and essential supplies [19].

#### Plan for change over time

The WHO guideline recommends that there should be a plan to monitor and evaluate CHW programmes and policies over time and to adapt and amend them through a dynamic process informed by context-specific evidence [19].

## Conclusions

Considering the significant contributions that CHW programmes are making to the delivery of local health services and the potential of expanded and improved CHW programmes to contribute to achieving the SDGs for health, UHC, ending preventable child and maternal deaths, and reducing health disparities, it is imperative to integrate CHW initiatives into formal health systems. Additionally, the multisectoral dimensions of CHW policy and programming can be better addressed through establishing and strengthening the national coordination mechanisms for HRH, and bringing in CHWs, CHW programmes, and their advocates as new, full-fledged partners in the process. This will enable related stakeholders to provide their CHW-related input by sharing their visions, engaging in policy dialogue, exchanging information, participating in joint decision-making, mobilizing resources, as well as cooperating in the implementation of CHW initiatives. Achieving this will require a strong national commitment to planning, coordination, and partnerships. Unfortunately, at present CHWs are far down at the very bottom of the HRH hierarchy and, in the minds of many, maybe not even a part of it. We do need to formalize their status as an essential and valued part of the health workforce and shift their positioning substantially. The effectiveness of planning, coordination, and formation of partnerships will depend on their recognition by and their formalized status within the health system.

## Data Availability

This paper is not based on primary or secondary data, and all external sources have been referenced.

## Abbreviations

AIDS: Acquired immunodeficiency syndrome
CCF: Country coordination and facilitation
CHW: Community health worker
COVID: Coronavirus disease
EQUINET: Regional Network on Equity in Health in East and Southern Africa
FLHWs: Frontline health workers
GHWA: Global Health Workforce Alliance
HRH: Human resources for health
MDGs: Millennium Development Goals
MERS: Middle East respiratory syndrome
MOH: Ministry of Health
NGO: Nongovernmental organization
NORAD: Norwegian Agency for Development Cooperation
SARS: Severe acute respiratory syndrome
SDGs: Sustainable Development Goals
SWAps: Sector-wide approaches
UHC: Universal Health Coverage
UN: United Nations
USAID: United States Agency for International Development

## Footnotes

1 The four consultations on CHWs and FLHWs in 2012 were as follows: (1) Technical Consultation on the Role of Community-based Providers in Improving Maternal and Newborn Health (30–31 May 2012), convened by the Royal Tropical Institute, in Amsterdam, the Netherlands), (2) Evidence Summit on Community and Formal System Support for Enhanced Community Health Worker Performance (31 May–1 June 2012), convened by the United States Agency for International Development (USAID) Global Health Bureau in Washington, DC), (3) Community Health Worker Regional Meeting (19–21 June 2012), convened by the USAID-funded Health Care Improvement Project in Addis Ababa, Ethiopia, and (4) Health Workers at the Frontline—Acting on What We Know: Consultation on How to Improve Frontline Access to Evidence-based Interventions by Skilled Health Care Providers (25–27 June 2012), convened by the Norwegian Agency for Development Cooperation (NORAD) and coordinated by the Regional Network on Equity in Health in East and Southern Africa (EQUINET) in Nairobi, Kenya.

2 The Three-Ones approach derives from an approach used in the AIDS response where countries had one national AIDS committee, one national plan, and one national monitoring and evaluation framework.
